# A proteasome inhibitor fails to attenuate dystrophic pathology in mdx mice

**DOI:** 10.1371/4f84a944d8930

**Published:** 2012-06-27

**Authors:** Joshua Selsby, Carl Morris, Linda Morris, Lee Sweeney

## Abstract

Dystrophin deficiency leads to increased proteasome activity in skeletal muscle. Previous observations suggest short-term inhibition of the proteasome restores dystrophin expression. Contrary to our hypothesis, eight days of MG-132 administration to mdx mice increased susceptibility to contraction induced injury and Evan’s blue dye penetration compared to controls. Following six weeks of MG-132 administration muscle function was similar to control animals. These data suggest that proteasome inhibition does not reduce the severity of muscle dysfunction caused by dystrophin-deficiency.

## Introduction

Duchenne muscular dystrophy is caused by a mutation in the gene coding for the dystrophin protein. The dystrophin protein links the actin cytoskeleton to the cell membrane through the dystrophin-glycoprotein complex (DGC) and ultimately to the extracellular matrix. In the case of DMD, the resulting gene product is not capable of performing this function and consequently the muscle fibers are more susceptible to contraction induced injury 1 2 3. DMD is characterized by periods of necrosis and regeneration with progressive fibrosis, proteolysis 4 5 , free radical generation 6 and apoptosis 7. The standard, and widely used, preclinical model is the mdx mouse 8.

Numerous strategies to blunt disease severity have been attempted and have met with mixed results. As proteolysis is consistently demonstrated to be increased in dystrophic muscle, protease inhibition has drawn a great deal of interest. For some time it has been appreciated that calpain activity is increased in dystrophic muscle 5. Despite early results to the contrary 9 10 , it now appears as though calpain inhibition does not improve dystrophic pathology 2 4. An alternative strategy has been targeted inhibition of the ubiquitin-proteasome pathway. Despite the potential risks posed by accumulating proteolysis cleavage products, these studies have shown improved histological measures as well as restoration of dystrophin and DGC component expression and localization 11 12 13 14. The purpose of this investigation was to determine the extent to which proteasome inhibition improved muscle function in mdx mice. Based on the results of prior studies 11 12 13 14 , we hypothesized that mdx mice treated with a proteasome inhibitor would have improved muscle function when compared to untreated mdx mice.

## Methods

All animal procedures were approved by the IACUC at the University of Pennsylvania and were done in accordance with the guiding principles established by the American Physiological Society. Male, four-week old mdx mice from our colony were injected with 10 µg/kg/day MG-132 (a proteasome inhibitor; n=5) or saline (n=8) i.p. for six weeks. An additional treatment group was only treated with MG-132 for the final eight days of the study period (n=5). Following the treatment period, animals were brought to a surgical level of anesthesia with ketamine/xylazine cocktail and the extensor digitorum longus (EDL) muscles were removed. Muscle function tests were performed in accordance with our previously described methods 1, 2, 3, 15. In addition, the diaphragm was removed for a determination of Evan’s blue dye permeability according to previously described methods 1, 2.

## Results

Short-term use of the proteasome inhibitor MG-132 increased absolute EDL mass by 8% (p<0.05) over untreated mdx controls though relative EDL mass (muscle to body weight) was similar between groups (Table 1). Absolute EDL mass was similar to control levels following six weeks of MG-132 use. Despite increased muscle mass associated with short-term MG-132 use, tetanic force was similar between the mdx and mdx MG-132 8 day groups. Longer-term MG-132 usage impaired tetanic force production by nearly 20% (p<0.05) compared to short-term use and was similar to untreated mdx mice. Cross-sectional area was increased in the 8-day MG-132 treated mdx mice compared to both the control mdx mice and the longer-term treated mdx mice (p<0.05), however, six weeks of use resulted in CSA that was 10% smaller than control (p<0.05). Changes in CSA were well matched with changes in tetanic force so specific tension remained similar between groups.

**Table 1:** Table 1. Selected parameters following MG-132 treatment. Mdx animals were injected with 10 ug/kg/day MG-132 ip for eight days or 6 weeks. * indicates significantly different from control (p<0.05). # indicates significantly different from MG-132 8 Day (p<0.05). Data is shown as mean ± SEM.

	***mdx Control***	*mdx MG-132 8 day*	*mdx MG-132 6 week*
Body Mass (g)	28.9 ± 0.6	31.3 ± 1.1	29.9 ± 1.0
EDL Mass (mg)	12.8 ± 0.2	14.7 ± 0.5*	12.3 ± 0.8#
EDL Mass/Body Mass (mg/g)	0.453 ± 0.01	0.474 ± 0.02	0.418 ± 0.03
Tetanic Force (mN)	406.8 ± 12.3	455.8 ± 17.0	373.1 ± 22.4#
CSA (mm^2^)	2.24 ± 0.04	2.55 ± 0.09*	2.00 ± 0.10*#
Specific Tension (mN/cm^2^)	18.1 ± 0.6	17.9 ± 0.7	18.7 ± 1.0
ECC Force Decrement (%)	-25.4 ± 2.5	-43.2 ± 4.7*	-28.2 ± 3.5#

Given previous results indicating increased dystrophin expression and restoration of the DGC with MG-132 administration, we also determined the extent to which MG-132 treatment would prevent contraction induced injury. Following five lengthening contractions eight days of MG-132 use impaired force production by approximately 40% (p<0.05) compared to untreated and 6 week treated mice though following six weeks of MG-132 use impaired force production was similar to untreated mdx mice. Because the mdx MG-132 8 day animals were more susceptible to contraction induced injury than untreated mdx mice we determined Evan’s blue dye penetration as an indicator of sarcolemma permeability. Muscles from the 8-day group were more susceptible to Evan’s blue dye penetration than control (Figure 1; 7.3% ± 1.0% mdx-Con, 17.9 ± 2.7% mdx-MG-132; n=3 /group) indicating increased membrane injury.

**Short-term MG-132 treatment increases Evan’s Blue Dye penetration into diaphragms.**
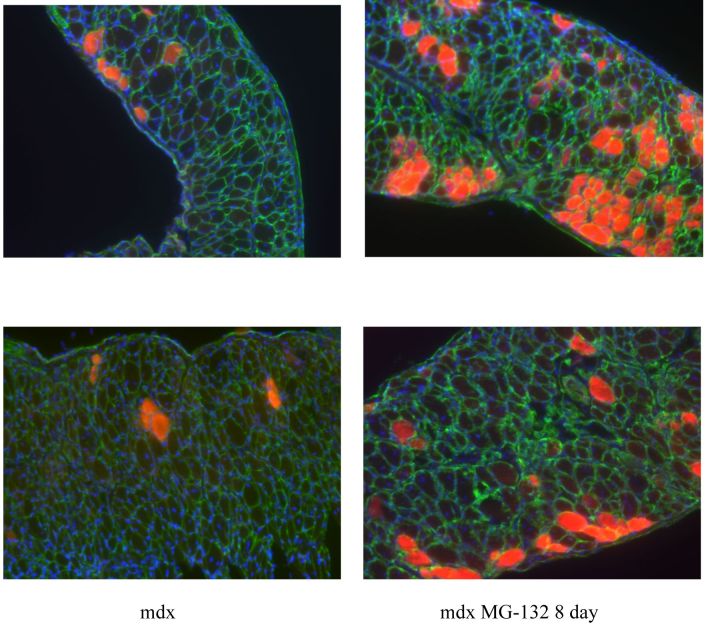
Nine week old mdx mice were injected daily with 10 µg/kg/day of MG-132 for eight days and the diaphragm removed. Quantification of EBD positive cells shows that MG-132 causes increased EBD penetration (17.9 ± 2.7%) into muscle cells compared to muscle taken from untreated mdx mice (7.3 ± 1.0%; n=3/group; p<0.05).

## Discussion

The purpose of this investigation was to confirm and extend previous observations that proteasome inhibition reduced disease manifestations in dystrophic muscle 11, 12, 13, 14. These previous studies showed short term treatment with MG-132 was able to improve the dystrophic phenotype in cell culture and mdx mouse models. It was unclear, however, whether chronic treatment would maintain the observed benefits and if reductions in muscle injury would translate into greater muscle function. In this study, mdx mice were treated with the proteasome inhibitor MG-132, for either eight days or six weeks. After eight days of treatment, several muscle parameters supported previous findings that MG-132 may be an effective treatment for DMD as muscle mass and tetanic force were increased. However, serious concern was raised when we demonstrated that muscles from these mice were more susceptible to contraction induced injury than untreated muscle. Moreover, histological analysis showed that eight days of MG-132 administration increased membrane permeability to EBD. Taken together, these data suggest possible toxicity and increased muscle damage with short-term MG-132 use. After six weeks of MG-132 administration improvements in functional parameters observed following eight days of MG-132 administration were lost, though muscle was no longer more susceptible to contraction induced injury. Proteasome inhibition does not appear to be beneficial to dystrophic muscle, and these data raise the possibility that it may exacerbate muscle dysfunction.

These data are in stark contrast to several previous reports 11, 12, 13, 14 in which it was found that proteasome inhibition resulted in restoration of expression and localization of dystrophin and DGC components in mdx mice and human explants. That these studies were so successful is surprising considering they show accumulation of only truncated dystrophin in western blots from muscles from either mdx mice or DMD patients 11, 12, 13, 14. Further, in mdx mice, the dystrophin gene product lacks the C-terminus, hence interaction with the DGC seems unlikely. Unfortunately, measurement of dystrophin and DGC expression and localization was beyond the scope of this investigation, however, to our knowledge, MG-132 does not suppress nonsense mutations indicating that accumulation of full length dystrophin and resultant restoration of DGC expression and localization would not be expected. Our data support this point as resistance to contraction induced injury was not improved by proteasome inhibition and was even impaired following eight days of MG-132 use. Moreover, our data demonstrate that reported histological benefits do not translate into functional benefits. Thus, while proteasome inhibition may provide short-term improvements in dystrophic muscle, continued inhibition appears to be deleterious or ineffective. Nevertheless, it is possible that intermittent dosing with a proteasome inhibitor could avoid potential adverse effects and find utility as a therapy in chronic diseases such as DMD.
